# Vulnerability to HIV of Internally Displaced Persons in the Republic of Serbia

**DOI:** 10.1186/1471-2334-14-S2-P34

**Published:** 2014-05-23

**Authors:** Miljana Grbic, Verica Lela Ilic, Sladjana Baros, Farida Bassioni Stamenic, Rade Grbic, Milan Parlic, Svetomir Samardzic

**Affiliations:** 1 United Nations Development Program, Belgrade, Serbia

## Background

Serbia hosts some 228,000 Internally Displaced Persons (IDPs) from Kosovo, of whom more than 2,000 live in 24 collective centers. A large number of refugees and IDPs reside in substandard temporary housing or in illegal settlements lacking basic facilities.

The research to assess vulnerability to HIV among young IDPs in Serbia was carried out with the support of the UN Joint AIDS Team. The goal of the research was to: assess the level and exposure to HIV risk behavior among young IDPs in two cities, Belgrade and Kragujevac, where the largest ID collective centers are placed; to inform stakeholders to tailor services to the needs of targeted population.

## Methods

Sampling was performed by combining methods of random choice of respondents using lists of registered young IDPs provided by UNHCR and the “snowball” method. Research instruments included procedure checklists and questionnaire, which consisted of 10 modules. The interviewers conducted filling-in of questionnaires. The sample included 305 IDP youth, with 31.1% of Roma youth which is in accordance with the estimated distribution of Roma in the overall IDP population.

## Results

The analysis has shown that accommodation of the respondents, together with their education, represented a significant variable in respect of risky behavior amongst IDP youth. The degree of knowledge about STI and HIV grows in parallel with better living conditions and higher level of education, as well as knowledge on where condoms can be obtained and the frequency of their use with permanent partners, while the frequency of pregnancies among young females decreases. Coverage with specific preventive programs is low (4.3%).

## Conclusion

Amongst the IDP youth, young Roma and those who have inferior living conditions show a greater degree of vulnerability to HIV in relation to those with better living conditions. Young females, in relation to young males, are at higher risk due to having less influence on the use of condoms during sexual intercourse.

